# How aging impacts skin biomechanics: a multiscale study in mice

**DOI:** 10.1038/s41598-017-13150-4

**Published:** 2017-10-23

**Authors:** Barbara Lynch, Christelle Bonod-Bidaud, Guillaume Ducourthial, Jean-Sébastien Affagard, Stéphane Bancelin, Sotiris Psilodimitrakopoulos, Florence Ruggiero, Jean-Marc Allain, Marie-Claire Schanne-Klein

**Affiliations:** 1 LMS, Ecole Polytechnique, CNRS, Université Paris-Saclay, Palaiseau, France; 2 Institut de Génomique Fonctionnelle de Lyon, ENS-Lyon, CNRS UMR 5242, Université de Lyon, Lyon, France; 3 LOB, Ecole Polytechnique, CNRS, Inserm, Université Paris-Saclay, Palaiseau, France; 4 Inria, Université Paris-Saclay, Palaiseau, France

## Abstract

Skin aging is a complex process that strongly affects the mechanical behavior of skin. This study aims at deciphering the relationship between age-related changes in dermis mechanical behavior and the underlying changes in dermis microstructure. To that end, we use multiphoton microscopy to monitor the reorganization of dermal collagen during mechanical traction assays in *ex vivo* skin from young and old mice. The simultaneous variations of a full set of mechanical and microstructural parameters are analyzed in the framework of a multiscale mechanical interpretation. They show consistent results for wild-type mice as well as for genetically-modified mice with modified collagen V synthesis. We mainly observe an increase of the tangent modulus and a lengthening of the heel region in old murine skin from all strains, which is attributed to two different origins that may act together: (i) increased cross-linking of collagen fibers and (ii) loss of water due to proteoglycans deterioration, which impedes inner sliding within these fibers. In contrast, the microstructure reorganization upon stretching shows no age-related difference, which can be attributed to opposite effects of the decrease of collagen content and of the increase of collagen cross-linking in old mice.

## Introduction

Aging is a complex process that affects the function of all organs and tissues and most often has an irreversible impact on their mechanical behavior. The most visible effects of aging are observed in skin and have been extensively studied for medical and cosmetic purposes. The three skin layers are affected both structurally and functionally. However, aging primary impacts the mechanical integrity of the dermis. At macroscopic scale, the mechanical behavior of aged dermis shows an increased stiffness and a decreased ability to recoil^1–3^. At lower scales, a complex multi-parameters process eventually results in a decrease of collagen and elastin contents due to an imbalance between matrix proteins synthesis and degradation by matrix metalloproteinases, an increase of collagen cross-linking, a deterioration of proteoglycans and a subsequent loss of water^4–9^. However, the link between these microstructural modifications and the mechanical changes has so far been inferred rather than experimentally demonstrated due to the technical issues encountered when trying to obtain multiscale data.

Collagens are the main component of the dermis and other connective tissues^7,10^. Fibril-forming collagens assemble into striated fibrils, the diameter and three-dimensional organization of which are tissue-specific. They form multiprotein networks with other matrix proteins such as the elastin fibers and non-fibrillar matrix (proteoglycans, glycoaminoglycans…) that determine the mechanical behavior of dermis and other collagen-rich tissues^11–18^. Collagen fibers are usually heterotypic structures. In dermis, they are made of type I, III and V collagens. Type V collagen is a minor component that acts as a regulatory fibril*-*forming collagen^19,20^. As such, it plays an important role in the pathogenesis of the classical Ehlers-Danlos (EDS) syndrome. This rare connective tissue disease illustrates the close link between collagen microstructure and tissue mechanics since it is caused by mutations in collagen V genes, while being primary characterized by skin hyperextensibility^19–21^. Moreover, EDS patients show a prematurely aged skin, which illustrates the close link between collagen microstructure and skin aging.

The relationship between collagen hierarchical structure and mechanical behavior has been explored using numerical simulations from the molecular scale^22–25^ and constitutive models have been proposed to explain the skin mechanical behavior^13–18^. Recently, multiphoton microscopy has been used to monitor the reorganization of collagen microstructure during mechanical assays in skin and in various tissues^26–37^. Fibrillar collagen indeed exhibits intrinsic Second Harmonic Generation (SHG) signals. Visualization of this signal in multiphoton microscopy allows to measure simultaneously the microstructural reorganization of the tissue under mechanical stimulation and the mechanical behavior at macroscopic scale, which provides multiscale experimental data not accessible using other techniques. We recently implemented this method on *ex vivo* skin samples from young adult mice^34^ and proposed a novel microstructural interpretation of skin biomechanics^37^. We further studied genetically-modified mice, focusing on collagen V, and compared wild-type (WT) mice to the transgenic *K14-COL5A1* ^38^ and knock-in *Col5a2* ^*pN/*+^ ^20,39^ mice.

This study aims at addressing the role of aging on the mechanical multiscale behavior of skin. This issue is addressed in murine skin because of easier availability of matched groups at different ages. We combined traction assays with multiphoton microscopy in *ex vivo* skin samples from mice aged 15 to 20 months (referred to as “old mice”). We compared these data to our previous results obtained in one-month old mice (referred to as “young mice”)^34^. We studied both WT and genetically-modified mice for which collagen V expression in skin has been modulated, inducing modified biomechanical behavior in young mice^20,34,37,38^. We observed differences in quantitative microstructural and mechanical parameters, which are discussed in the framework of our recently proposed microstructural interpretation of skin mechanical behavior.

## Results

**Figure 1:**
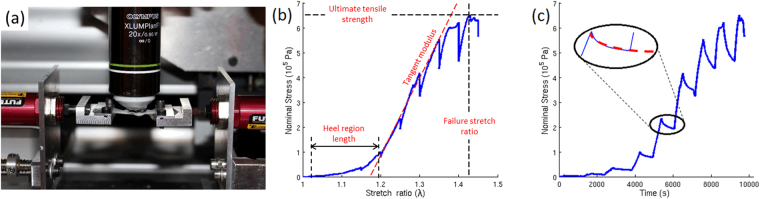
Mechanical assays under SHG microscope. (**a**) Side view of the setup, showing the skin sample attached to the traction device by use of metallic jaws. Immersion gel prevents skin dehydration and ensures optical contact with the objective lens. (**b**) Nominal stress/stretch curve obtained for an old WT mouse, showing the parameters used to quantify the macroscopic mechanical response. Blue solid curve: experimental data; red dashed curve: linear fitting providing the tangent modulus. (**c**) Nominal stress/time curve for the same sample showing series of short relaxations when the stretching is stopped during SHG imaging. Inset: zoomed relaxation, showing fitting with Eq. (1) (red dashed curve).

**Figure 2:**
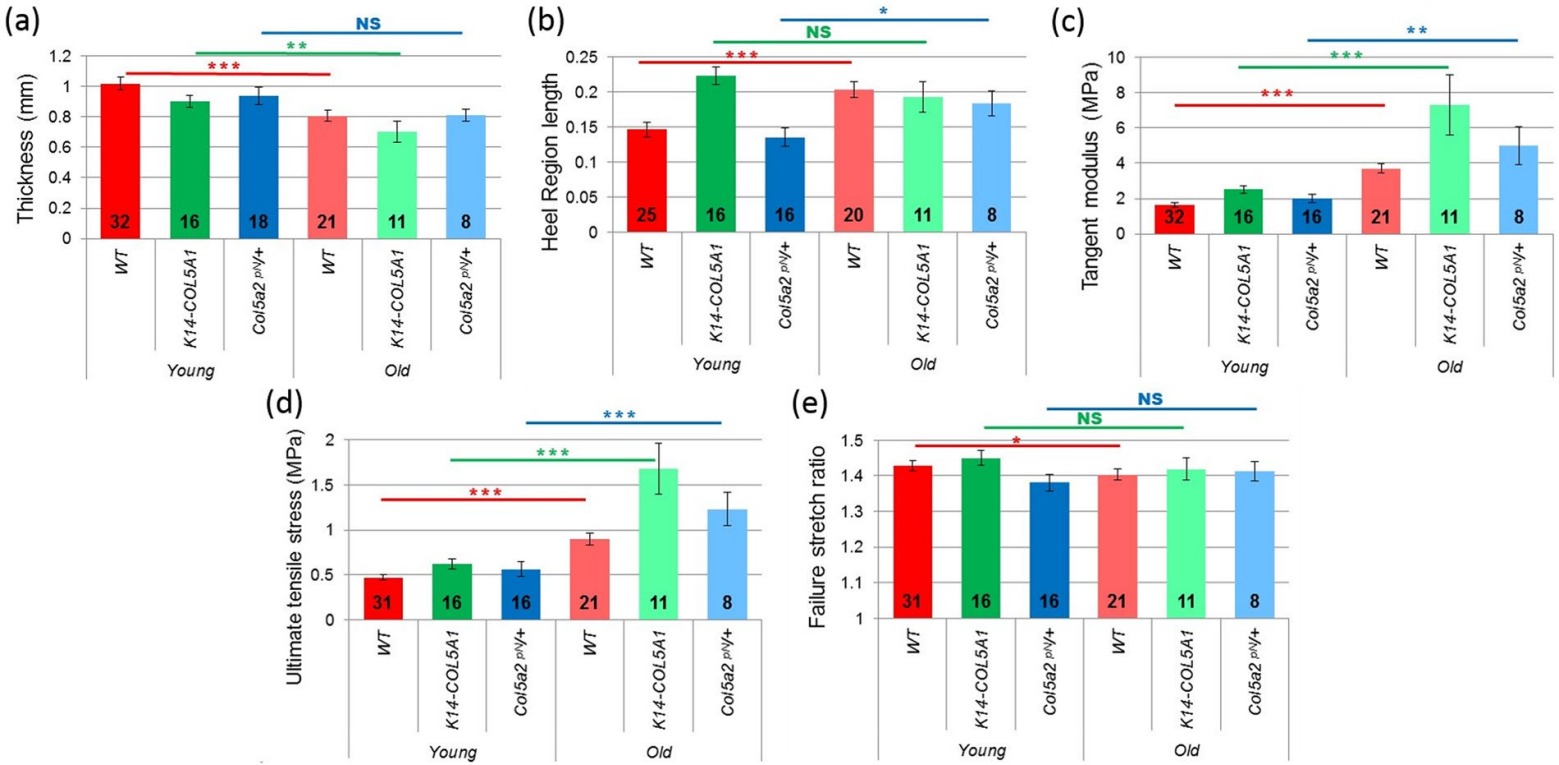
Mechanical data for young (dark colors) versus old (pale colors) WT *(red), K14-COL5A1* (green) and *Col5a2* ^*pN/*+^ mice (blue). (**a**) Skin thickness, (**b**) Length of the heel region, (**c**) Tangent modulus, (**d**) Ultimate tensile stress, (**e**) Failure stretch ratio. The number of mice in every set of data is indicated on each bar. Error bars correspond to SEM. NS: non-significant, *p < 5%, **p < 1% and ***p < 0.1%.

We performed uniaxial traction assays under multiphoton microscope in depilated and de-epidermilized *ex vivo* skin samples collected from the back of WT*, K14-COL5A1* ^38^ and *Col5a2* ^*pN/*+^ ^20,39^ mice aged 15 to 20 months (Fig. 1a). All these data were measured and processed in the exact same way as our previously reported data for one-month old mice in order to enable reliable comparison between young and old mice^34,37^. Four types of data were obtained simultaneously: (i) the nominal stress (hereafter called “stress” for readability) was measured as a function of the imposed stretch ratio (macroscopic mechanical response); (ii) the relaxation occurring during the short time periods when the traction was stopped for multiphoton imaging was also analyzed; (iii) the reorganization of the microstructure was characterized using SHG microscopy to visualize collagen fibers; (iv) local deformation data were also obtained from the deformation of the hair follicles network. In addition, the initial thickness of the skin samples was measured. It decreased with age in all strains (Fig. 2a and Supplementary Figure S1a). The decrease was significant for WT and *K14-COL5A1* mice, but not for *Col5a2* ^*pN/*+^ mice (probably because of the lower number of old mice from this strain).

### Stress/stretch behavior

Qualitatively, the macroscopic mechanical behavior of aged mice skin was similar to the response of young mice skin^34,37^. The stress/stretch relationship followed the classical J-shaped curve with toe, heel, linear and rupture regions (Fig. 1b). Quantitative differences were observed for the 4 parameters presented in Fig. 1b. Comparative data are summarized in Fig. 2 and relative data (ratio of average for old mice to average for young mice) are shown in Supplementary Figure S1. The length of the heel region significantly increased for WT and *Col5a2* ^*pN/*+^ mice, and did not change for *K14-COL5A1* mice. The tangent modulus increased for all murine strains by a factor ranging from 2.3 (WT) to 2.9 (*K14-COL5A1*) between young and old mice. The ultimate tensile stress exhibited the same behavior, with a slightly smaller increase. Finally, the failure stretch ratio did not vary significantly, except for WT mice, where it slightly decreased by a factor 0.98.

### Relaxation analysis

**Figure 3:**
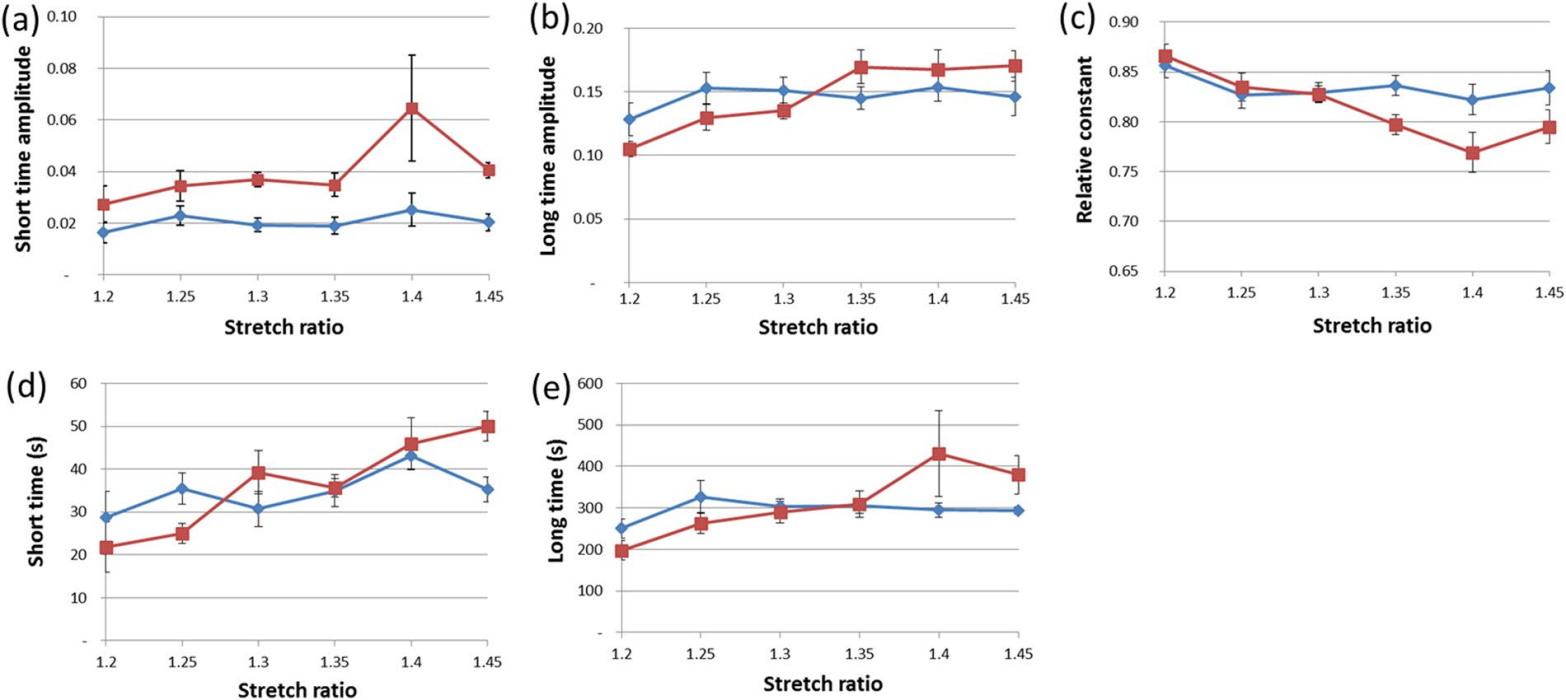
Relaxation data for young (blue) versus old (brown) WT mice. Normalized amplitudes of the (**a**) short-time, (**b**) long-time, and (**c**) constant components of the relaxation curves when fitted with Eq. 1. Values of the (**d**) short and (**e**) long relaxation times. Error bars correspond to SEM.

In experiments under SHG microscope, the sample relaxed when we stopped the motors for SHG imaging (Fig. 1c). We analyzed these short time relaxations after normalization in order to enable comparison of normalized stresses. We fitted the normalized data using two exponential functions and a saturation constant, and obtained the five parameters displayed in Fig. 3 for young and old mice at different stretch levels. The three relative relaxation magnitudes are not independent as their sums have to be set at 1 because of the normalization. We only analyzed relaxations in the linear part of the stress/stretch curve for WT mice because the number of samples was too low in other cases. We observed little changes in the relative relaxation behavior at low stretches between old and young mice. The relative relaxations appeared to be slightly faster in old mice, but the saturation constant – which includes all the longer relaxations effect – was not affected by age. The relative relaxation parameters for old mice evolved with the stretch level, contrary to the ones for young mice. The short and long time amplitudes obtained from the relaxation fitting analysis increased with stretch, as did the short and long relaxation times. As a consequence, the saturation constant decreased. Thus, as the skin was increasingly stretched, the relative relaxation became slightly faster.

### Microstructural analysis

**Figure 4:**
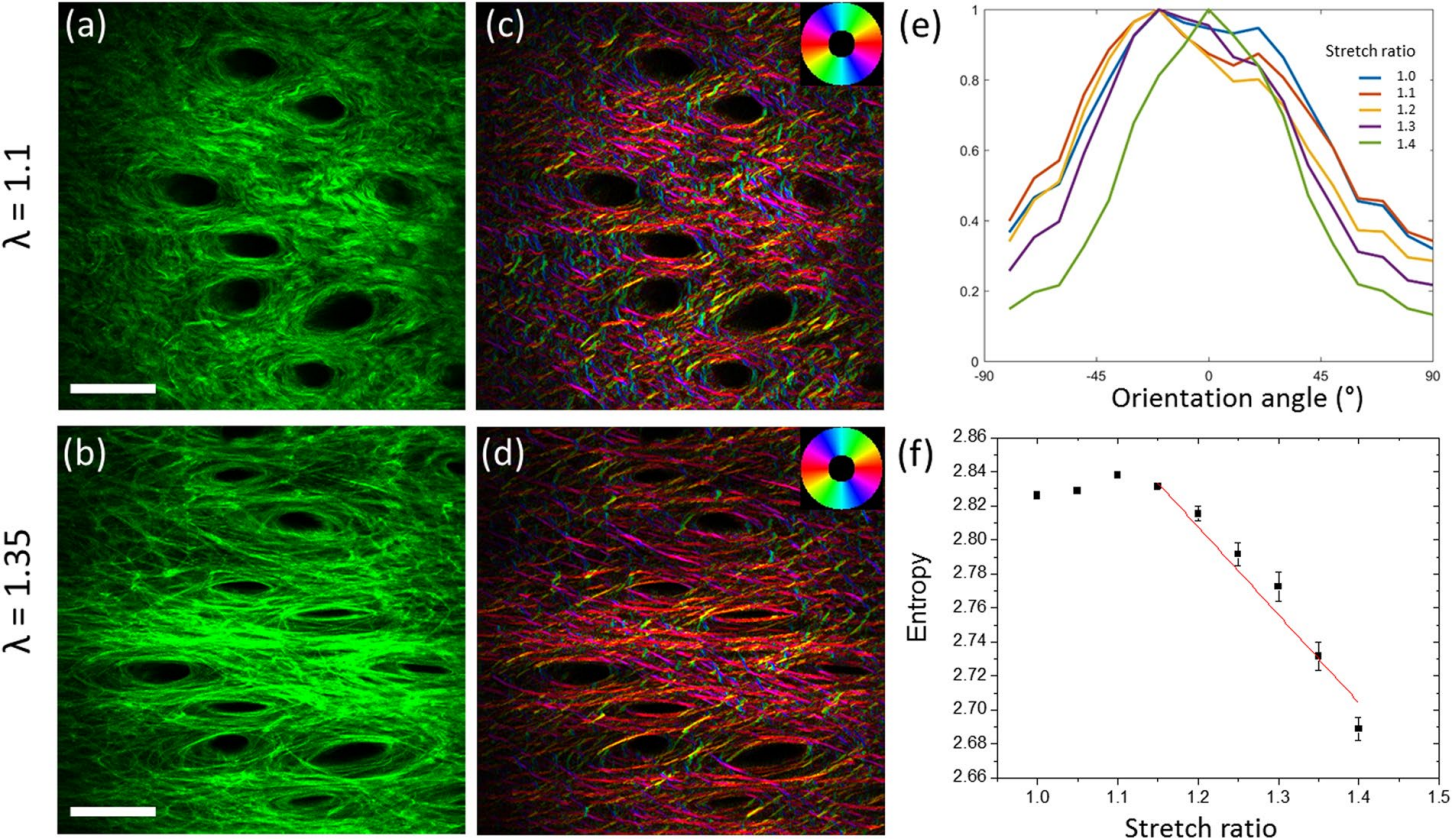
SHG imaging of collagen reorganization in skin sample from an old WT mouse (same as in Fig. 1b and c). (**a**,**b**) SHG images of an old WT mouse skin at (**a**) 1.1 and (**b**) 1.35 stretch ratio, showing a network of collagen fibers. The positions of hair follicles are revealed as dark regions with no SHG signal. The traction is along the horizontal direction. Scale bar: 100 µm. (**c**,**d**) Orientation mapping of collagen fibers extracted from the SHG images. The color code is given in the insert. (**e**) Orientation histograms of the collagen fibers obtained from the SHG images at increasing stretch ratios (only 1 out of 2 histograms is displayed). (**f**) Variation of the entropy of the orientation histogram as a function of the stretch ratio. The red line corresponds to a linear fitting.

SHG imaging revealed collagen fibers in the dermis, around elliptical regions with no signal that corresponded to hair follicles (Fig. 4). SHG imaging at increasing stretch ratio therefore enabled to characterize changes of skin microstructure. The microstructural behavior for old mice of all strains was qualitatively the same as for young mice^34,37^. The collagen fibers oriented gradually in the direction of traction when the skin sample was stretched, while the hair follicles became more and more elliptical along the traction axis. The initial distribution of fibers in old mice exhibited either two peaks, typically at 20° to 40° to the anterio-posterior axis, or a unique wide peak, which evolved into a thinner single peak distribution after the heel region (Fig. 4e). Finally, the orientation index (OI) obtained from this distribution followed the stress curve, similarly to what was described for young mice^34,37^: it increased linearly with stretch after the heel region, while the entropy of the orientation distribution decreased linearly in the same region (Fig. 4f).

**Figure 5:**
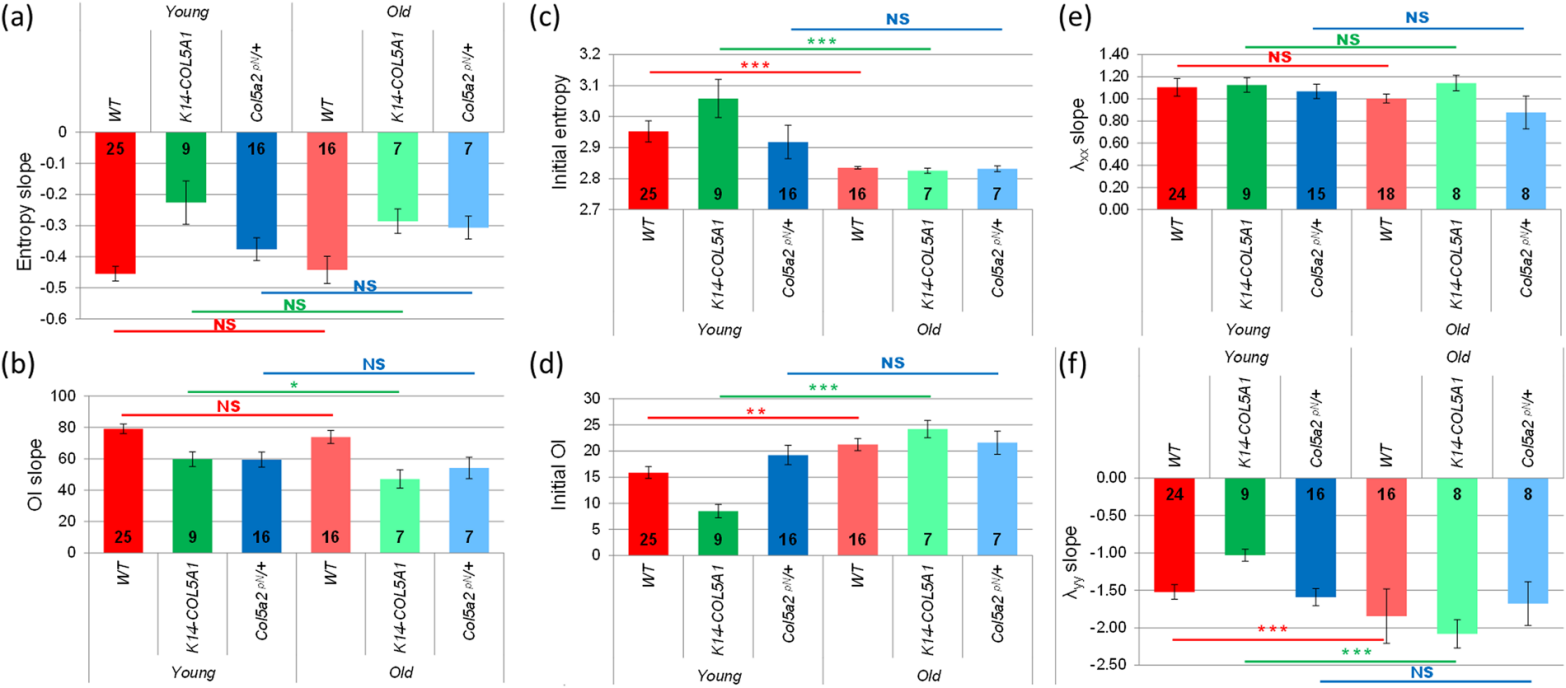
Microscopic data for young (dark colors) versus old (pale colors) WT (red), *K14-COL5A1* (green) and *Col5a2* ^*pN/*+^ mice (blue). Slope of the linear region of (**a**) the entropy variation and (**b**) the OI variation as a function of the stretch ratio. Initial value (no applied stretch) of (**c**) the entropy and (**d**) the OI. Slope of (**e**) λ_xx_ variation and of (**f**) λ_yy_ variation as a function of the stretch ratio. Error bars correspond to SEM. The number of mice in every set of data is indicated on each bar. NS: non-significant, *p < 5%, **p < 1% and ***p < 0.1%.

Quantitative comparison of the microstructural behavior of young and old mice is summarized in Fig. 5 and Supplementary Figure S2 (relative data). The slopes of the linear part of the OI and of the entropy showed no significant difference for WT and *Col5a2* ^*pN/*+^ strains, and only a small decrease for the OI of *K14-COL5A1* mice. In contrast, the initial OI and entropy measured before stretching exhibited significant differences for all strains but *Col5a2* ^*pN/*+^: the initial entropy decreased, while the OI increased.

### Local deformation analysis

The hair follicle network appearing as dark spots in SHG images was used to compute the local deformation of the skin sample at the scale of a few hundreds of micrometer (Fig. 4 and Supplementary Figure S3). The local stretch ratio in the direction parallel to the traction, λ_xx,_ increased linearly when the applied deformation increased. The slope of this linear increase was consistently close to 1 for all mice (Fig. 5e and Supplementary Figures S3c and S4a). This showed that the skin behavior was homogeneous and that the skin sample was properly blocked within the jaws, without any slippage, which validated our macroscopic stretch measurements. The local stretch ratio in the direction perpendicular to the traction, λ_yy_, first slightly increased for small applied stretch ratios and then decreased linearly when the applied deformation increased. The same behavior was observed for young mice^34^. The initial increase may be due to a poroelastic effect, favored by the absence of epidermis, while we don’t have a definite explanation for it. The absolute value of the slope of the linear region increased significantly for old mice of WT and *K14-COL5A1* strains, while it showed no difference between young and old *Col5a2* ^*pN/*+^ mice (Fig. 5f and Supplementary Figure S4b).

## Discussion

Age-related microstructural changes in the dermis affect collagen fibers as well as the other components of the extracellular matrix^2–5^. Notably, there is a progressive decrease in collagen, elastin and proteoglycans content, an increase in glycation cross-linking within and between fibers and the matrix proteins become fragmented with little spatial structure. Accordingly, the dermis is thinner, as reported in the literature for human skin^40^ and observed in our data for murine skin (Fig. 2a). The age-related structural changes of the skin have been reported to be largely similar in human and murine skin^9^. Specifically, it has been reported that the collagen content decreases by about 30% between 2 (young) and 22 (old) months in murine skin^41^. However, our findings in murine skin may not be fully valid for human skin.

These age-related structural changes are expected to affect the multiscale mechanical behavior of skin. We recently investigated the complex link between microstructure and mechanical properties in young mice skin. We proposed an interpretation based on three different mechanisms^37^, that remains for the moment descriptive. It relates the micrometer scale (i.e. the scale of the collagen fibers) to the tissue scale. The smaller scales of the hierarchical structure of the collagen molecules are included in the effective fiber behavior. In the toe and heel regions, no significant evolution of the fibers orientation was observed, and low values of the stress were measured. The mechanical response in this region was associated to the response of the collagen network as a structure. It is controlled by the buckling of the collagen fibers perpendicular to the traction direction and the bending of the reticulation points. The non-fibrillar matrix also contributes as a visco-elastic material and prevents any motion of the network. In the linear region, the fibers align in the direction of traction, proportionally to the stretch. The linearity of the stress/stretch relationship is then explained by a plastic response of the collagen fibers that is attributed to sliding between fibrils connected by proteoglycans, inside the fibers. The surrounding matrix plays also a role in both the alignment capability and the local plasticity of the collagen fibers.

It is important here to emphasize the non-affine behavior of the fiber network in the linear part of the stress-stretch curve^35^. An affine behavior means that the evolution of the fibers network can be directly predicted from the motion of the volume in which they are embedded. If this were the case, the OI evolution would contain the same information as the evolutions of λ_xx_ and λ_yy_. So, the effect of aging wouldn’t be measurable with our approach. As the OI evolution differs significantly from the affine response, our microscopic observations are likely to be affected by the microstructural consequences of aging. Indeed, while the multiscale behavior of old mice skin is qualitatively comparable to the one of young mice skin (Fig. 1), quantitative differences are observed for some parameters (Fig. 5), which we attribute to aging effects. In the discussion of these differences, it is important to keep in mind that our results were obtained from *ex vivo* experiments, which cannot reflect perfectly the real, *in vivo*, state of the skin, and especially its pre-stress.

**Figure 6:**
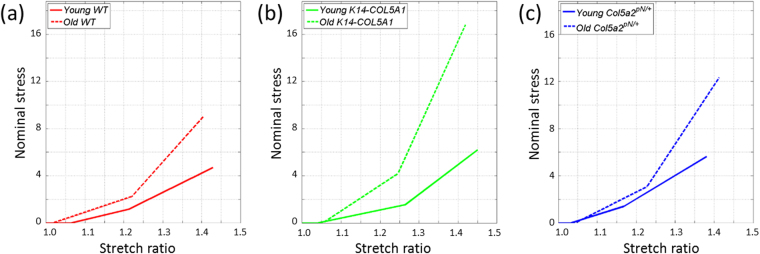
Simplified scheme of Nominal stress / Stretch ratio curves for young versus old WT*, K14-COL5A1* and *Col5a2* ^*pN/*+^ mice. Young WT: Red solid line, young *K14-COL5A1*: green solid line, young *Col5a2* ^*pN/*+^: blue solid line; old WT: Red dashed line, old *K14-COL5A1*: green dashed line, old *Col5a2* ^*pN/*+^: blue dashed line.

Regarding mechanical data in WT skin (Fig. 2), we observed a variation of most parameters, as summarized in Fig. 6, which shows trends curves based on our measurements and allows a visual direct comparison of the variations of skin mechanical behavior with age. First, the slight decrease of the sample initial thickness is consistent with the decrease of collagen content expected from the literature^40^. This was too small to explain the other variations in mechanical behavior. Second, the lengthening of the heel region can be explained on the basis of our multiscale mechanical interpretation by the stiffening of the fibers and by the increased connectivity of the collagen network due to age-related crosslinks. These two effects prevent the buckling of the collagen network, and thus lengthen the stretch needed for its reorganization. The surrounding non-fibrillar matrix may block the transverse motions of the collagen fibers, and thus its degradation with age should work the opposite way, simplifying the network rearrangement and thus decreasing the heel length. This effect does not appear to be dominant here. The lengthening of the heel region may also originate in the damage of elastic fibers. Further inspection of this hypothesis would require data on purified elastin fibers, while only purified elastin from aorta that still exhibits the very specific laminae structure has been studied up to now^42,43^, which cannot be directly applied to a loose fiber network as in skin. Third, the increase in the tangent modulus, which is consistent with the increased stiffness reported in the literature^1–3,44^, can be attributed in our interpretation to two phenomena: (i) the increased cross-linking between fibers results in an increased stiffness of the collagen network; (ii) the degradation of proteoglycans leads to a loss of water and impedes sliding of fibrils inside the fibers, which results in a larger inner shear and in an increased plastic stress per fiber. Fourth, the observed increase of the ultimate tensile stress in old mice can be imputed to the same mechanisms. Finally, the slight decrease of the failure stretch ratio implies that the lengthening of the heel region is compensated by a shortening of the linear region. This means that the fibers are easier to break, which is consistent with the increased cross-linking between fibers, which decreases the sliding capability of each fiber. Easier breaking is also favored by the shortening of the fibrils in the fibers, which will then separate more easily.

The results of relaxation analysis of WT mice (Fig. 3) showed very close relative relaxation parameters for old and young mice. This implies that the dissipative mechanisms are likely similar: the inner-fiber sliding and the friction between fibers and surrounding proteoglycans. This may seem to contradict the increase in the tangent modulus. It implies that the viscous dissipations increase as much as the elastic stiffnesses, so that their ratio remains constant, as measured by relative relaxation. The variations of the relaxation parameters of old mice with stretch may be explained by an earlier development of damage in the fibers. This damage decreases the stiffness of the fibers, and thus favors a low saturation constant and larger relaxation times. A consequence of this accelerated creep relaxation is that the skin is likely to recover less easily after a given deformation. This could be tested through cyclic loadings. Though they are difficult to perform under microscope for relaxing membrane, these experiments could be directly compared to previous reports^45^.

Microstructural data for WT mice (Fig. 5) showed that the microstructural reorganization upon imposed stretch (OI and entropy slope) is the same for young and old mice. This might be surprising considering the many age-related changes in the skin ultrastructure and the quantitative changes of the mechanical parameters. This absence of variation may be explained by the combination of two effects that compensate each other: the increase of cross-linking in old mice impedes the collagen network reorganization, while the decrease of collagen content facilitates this reorganization. Most importantly, the initial values of the OI and entropy are significantly different in old mice compared to young mice. The OI increases and the entropy decreases, which means that the collagen network has a higher level of organization in old mice, in agreement with previous study^9^. This may be attributed to the cumulated effect of skin stretching during lifespan, which is facilitated by the reduced content of collagen and its increased cross-linking. Obviously, this initial configuration is not the *in vivo* configuration as the skin was detached (releasing inner stresses) and stored in culture medium: it is likely that the physiological state is even more anisotropic. Still, these differences can be considered as reminiscent of existing ones in *in vivo* skin.

Finally, a significant increase of │λ_yy_│ slope was observed in old mice (Fig. 5), which means that the overall volume of old skin samples decreased upon stretching compared to young mice. This may be explained by the degradation of proteoglycans in old mice, which results in a decreased efficiency to retain water during mechanical stimulation, and therefore a smaller final volume.

The link between microstructural and mechanical changes in old mice was further challenged by recording multiscale data in mice with modified skin microstructure^34^. The *K14-COL5A1* mice have been designed to force the expression of the homotrimeric collagen V in keratinocytes. Collagen V homotrimers do not form heterofibrils with collagen I and accumulate in papillary dermis as very thin collagen V fibrils^38^, which increases the viscosity of the non-fibrillar matrix^34^. The exon 6 deletion in *Col5a2* gene in the *Col5a2* ^*pN/*+^ mice favors the expression of the homotrimeric collagen V and results in larger and fewer collagen I fibers. TEM observations revealed, in the *Col5a2* ^*pN/pN*^ dermis^20^, a substantial increased quantity of proteoglycans that filled the space between the rare fibers, while this increase could not be analyzed quantitatively. In this study we used the heterozygous mice and not the homozygous mice as in ref.^20^, so that these changes may be lowered. Other differences between these strains, for instance different proteoglycan content or different quantity of collagen crosslinks, cannot be excluded and may affect the mechanical properties, mainly the viscosity of the non-fibrillar matrix.

The multiscale mechanical behavior of the old genetically-modified mice was qualitatively similar to the one of WT mice, as previously reported for young mice^34^. The age-related quantitative changes were also similar to the ones observed in WT mice, except for a few parameters (Fig. 6). Regarding *K14-COL5A1* mice, the main difference was found for the length of the heel region: it showed no significant changes in old mice, while a significant increase was observed for WT mice. This effect may be explained by the higher length of heel region for *K14-COL5A1* young mice, which could not increase further in old mice. The absence of significant evolution of failure stretch ratio for *K14-COL5A1* old mice was not surprising given the slight evolution for WT mice and the smaller statistics for *K14-COL5A1* mice. In the same manner, the evolution of the OI slope was only slightly significant and barely different from the one for WT mice. More striking differences were observed in the behavior of *Col5a2* ^*pN/*+^ mice. Only a slight difference was observed in the failure stretch ratio, as for *K14-COL5A1* mice, so that the macroscopic behavior of this strain was very similar to the one of WT. Contrary to WT and *K14-COL5A1* mice, we observed no significant change in the initial OI and entropy value and in the slope of │λ_yy_│ for old *Col5a2* ^*pN/*+^ mice compared to young *Col5a2* ^*pN/*+^ mice. This means that the collagen microstructure of *Col5a2* ^*pN/*+^ mice skin does not exhibit significant age-related changes. This surprising observation may be attributed to the increased quantity of proteoglycans in *Col5a2* ^*pN/*+^ mice. Firstly, it results in a more viscous non-fibrillar matrix that may impede the age-related increase in organization level of the collagen network and strongly limit the initial OI increase and entropy decrease. Secondly, it may compensate for the age-related decrease of proteoglycan content, and thus allow old mice skin to have the same ability to retain water as young mice skin, resulting in the same slope for │λ_yy_│. Further discussion of these differences between strains would require a thorough quantification of collagen crosslinks and proteoglycans content, which is beyond the scope of the present study.

In conclusion, our simultaneous observations of the mechanical behavior at macroscopic scale and of the microstructure of dermis are well explained in the framework of our multiscale interpretation of skin mechanics, which seems applicable to both aged and young murine skin. The two main microstructural changes affecting the mechanical properties appear to be the age-induced cross-linking and the degradation of the proteoglycan non-fibrillar matrix, which let the water flow out more easily. Together, they explain the lengthening of the heel region and the striking increase in tangent modulus in WT mice and mice with modified collagen V synthesis. Surprisingly, macroscopic relaxation and microscopic reorganization of the collagen network upon stretching do not change with age. It may be attributed to the decrease of collagen content compensating the increase of collagen cross-linking, thus preserving the molecular mechanisms creating inner- and inter-fibers relaxations. Only the initial level of organization of this collagen network is increased in aged mice, except for *Col5a2* ^*pN/*+^ mice presumably due to the increased content of proteoglycans inducing a higher tissue viscosity. All these considerations emphasize the complex role of the microstructure in the mechanical properties. Our findings open the door to future research on human skin to verify whether the above findings obtained in murine skin fully apply to human skin.

## Materials and Methods

### Murine skin samples

Wild-Type mice were compared to two transgenic murine models obtained from the same litters: the transgenic *K14-COL5A1* mouse line, which overexpresses the human proα1(V) under the control of the keratin 14 promoter^38^ and the mouse model for classic EDS with an in-frame exon 6 deletion in *Col5a2* (*Col5a2* ^*pN*^^*/*+^)^20,39^. Mice were sacrificed by cervical dislocation either at one month (66 young mice) or at 15–20 months (41 old mice), and studied using either multi-scale assays, SHG data only (in case of technical issues in the mechanical measurements), or mechanical measurements only (Supplemental Table 1). In order to limit the number of sacrificed mice with regards to the 3 R principles of research ethics, all the young mice data are identical to the ones previously reported^34^.

Skin samples were collected using the same protocol as previously described^34^. Briefly, skin of the back was depilated and de-epidermilized and the right foreleg was spotted with black ink to identify the anterio-posterior axis. Skin samples were stored in culture medium (Dulbecco’s Modified Eagle’s Medium, Sigma-Aldrich) without phenol red, supplemented with 50 µg/mL penicillin/streptomycin (Sigma) at 6 °C and used within five days for the biomechanical experiments. Ear biopsies were systematically collected for genotyping analysis.

All animal experiments were performed under animal care procedures and conducted in accordance with the guidelines set by the European Community Council Directives (86/609/EEC). All experimental procedures were approved by the Direction of the Veterinary Service of Rhone Department (DDSV, Lyon, France).

### Histology and immunochemistry

Total skin from WT, *K14-COL5A1* and *Col5a2* ^*pN/*+^ mice were fixed in 4% PFA, embedded in paraffin, and 5 μm thick sections were performed. Deparaffined sections were then stained with the Masson’s trichrome Goldner method (MTG) or incubated with polyclonal antibodies against elastin (IHC) (Novotec, Lyon, France). These results are presented in Supplementary Figure S5.

### Multiscale mechanical assays

Multiscale mechanical assays were performed as previously described^34^. Briefly, skin samples were cut into a dog-bone shape to ensure homogeneous uniaxial tensile load in the central testing portion (See Fig. 1). They were attached to a custom-built uniaxial traction device by use of metallic jaws, and inserted in place of the stage of a custom-build multiphoton microscope. The traction was applied in the anterio-posterior direction, with the papillary dermis up facing the objective lens, without any preconditioning. Immersion gel (Lacrygel, Europhta) was used to ensure optical contact with the objective lens and to prevent skin dehydration during experiments. Symmetric stretching at a slow strain rate (10^−4^ s^−1^, typically 2 µm.s^−1^) was imposed to the skin to monitor the same Region of Interest (ROI) during all the experiment, as verified by the characteristic pattern of hair follicles in the SHG images, and the resulting force was measured every second. Stretching was stopped during multiphoton imaging to avoid detrimental skin movements, resulting in an incremental loading path with steps of 0.05 stretch ratio. Multiphoton signals were excited by a femtosecond laser set at 860 nm (Mai-Tai, Spectra-Physics), using 30 mW typical power at the focus of a 20×, 0.95 NA objective lens (Olympus). Typically, 480 × 480 × 50 µm^3^ image stacks were epi-collected at 100 kHz pixel rate, using 0.5 µm pixel size and 2 µm axial steps (5 minutes recording time). These image stacks probed the papillary dermis and the upper reticulary dermis, with no visible abrupt limit between these two compartments. The duration of such incremental loading assays with multiphoton imaging was typically 3 hours until breakage of the skin. Some complementary mechanical assays were performed outside the multiphoton microscope in order to check that relaxation during short pauses for SHG imaging did not affect the general mechanical behavior. These assays were similar to the ones under the SHG microscope and their results were merged to the other mechanical results in Figs 2 and S1.

### Mechanical data processing

The global stretch ratio λ was obtained as the imposed length between the jaws divided by the initial length^34^. The initial (or reference) configuration was defined as the position where no vertical displacement (within 3 µm) was observed anymore when stretching the skin sample with continuous SHG imaging. The nominal stress was obtained as the measured force divided by the initial skin section, which was measured by use of a digital caliper. The absolute value of these parameters was not precisely determined because of the measurement uncertainty in the skin initial dimensions, but their variations were accurately determined.

We determined 4 parameters from the nominal stress/stretch data as previously described^34^: (i) the length of the heel region, (ii) the tangent modulus, obtained as the slope of the linear part of the curve, (iii) the ultimate tensile stress, obtained as the maximum stress before breakage, and (iv) the failure stretch ratio, corresponding to this stress (see Fig. 1b).

We also quantified the relaxations occurring during the short time periods when the traction was stopped for SHG imaging (see Fig. 1c), as previously described^37^. To obtain relative quantities and enable comparison at various stress levels, we divided the nominal stress by the initial nominal stress before relaxation, St(0), for every relaxation on the nominal stress/time curves. We then fitted all these short time relaxation curves with a bi-exponential function with saturation:

1 $$\frac{St(t)}{St(0)}={a}_{1}\cdot {e}^{-t/{t}_{1}}+{a}_{2}\cdot {e}^{-t/{t}_{2}}+{s}_{0}$$

This relaxation analysis eventually provided five parameters: the relative amplitude a_1_ and the relaxation time t_1_ for short time relaxation, the relative amplitude a_2_ and the relaxation t_2_ time for long time relaxation, and the relative constant for the relaxation s_0_, which was dependent on the two other amplitudes: a_1_ + a_2_ + s_0_ = 1. This analysis was performed only in the linear part of the stress curve because of too low signal to noise ratio in the heel region, and only for WT mice because of too low sample number for genetically-modified mice.

### SHG image processing

Series of SHG images at increasing stretch ratio provided 2 types of information. Firstly, the round regions with no SHG signal, corresponding to hair follicles, were used as endogenous tags to measure the deformation of the ROI with respects to the initial image with no stretch^34^ (see Figure S3). Three parameters were obtained: the local stretch ratios in the direction parallel λ_xx_ and perpendicular λ_yy_ to the traction, and the sliding angle, representing the shear and usually negligible. The evolution of λ_xx_ and λ_yy_ as a function of the applied stretch ratio showed a linear region and the slopes of these linear regions were computed for every mouse.

Secondly, the local orientation of the fibers revealed by the SHG images was extracted in every pixel using a custom-written Matlab script based on mathematical morphology algorithms^34^. It provided a histogram of the fiber orientation in the imaged region as a function of the stretch ratio. We then quantified this distribution of orientations using 2 parameters^34,46^: (i) the Orientation index (OI), defined as the percentage of fibers oriented along the main orientation, and (ii) the entropy, defined as the usual statistical entropy, independently of any main orientation:

2 $$S=\sum _{\theta =-{90}^{^\circ }}^{{90}^{^\circ }}p(\theta )\mathrm{ln}[p(\theta )]$$

where

3 $$p(\theta )=\frac{I(\theta )}{\sum _{\theta =-{90}^{^\circ }}^{{90}^{^\circ }}I(\theta )}$$

Both parameters showed a roughly flat region followed by a linear region as a function of the stretch ratio. We then extracted for both parameters the initial value for unstretched skin (average value in the initial flat region) and the slope of the linear region. Note that for the initial value, entropy is a more reliable parameter because the orientation histogram of unstretched skin often exhibits two main orientations.

### Statistics

Error bars correspond to Standard Error of the Mean, except for relative data obtained as ratio where they correspond to Standard Deviation (Supplementary figures). Statistical tests were performed using R (R development core team, R foundation for statistical computing). Using a 1% threshold, we first verified the normality of the distribution using the Shapiro-Wilk test as well as the equality of variance with the Fisher test. In case of normality, the significance of the mean difference was assessed using a sided two-sample t-test for equal variances, while we used the Welsh t-test modification otherwise. Finally, for non-normal distributions we determined the significance using a sided Wilcoxon test.

### Data availability

The authors declare that materials, data and associated protocols are available upon request to M.-C. Schanne-Klein: marie-claire.schanne-klein@polytechnique.edu.

## Electronic supplementary material

Supplementary information
