# Supplementary material for: How aging impacts skin biomechanics: a multiscale study in mice
Source: Sci Rep. 2017 Oct 23;7:13750. doi: 10.1038/s41598-017-13150-4 (PMC5653787; doi:10.1038/s41598-017-13150-4)
Supplement: Supplementary file 1 — Supplementary information [file 41598_2017_13150_MOESM1_ESM.pdf]

SUPPLEMENTARY INFORMATION

HOW AGING IMPACTS SKIN BIOMECHANICS: A MULTISCALE STUDY IN MICE.

Barbara Lynch#(1), Christelle Bonod-Bidaud#(2), Guillaume Ducourthial(3), Jean-Sébastien Affagard(1,4), Stéphane Bancelin(3), Sotiris Psilodimitrakopoulos(3), Florence Ruggiero*(2), Jean-Marc Allain*(1,4), Marie-Claire Schanne-Klein*(3)

(1) LMS, Ecole Polytechnique, CNRS, Université Paris-Saclay, Palaiseau, France

(2) Institut de Génomique Fonctionnelle de Lyon, ENS-Lyon, CNRS UMR 5242, Université de Lyon, Lyon, France

(3) LOB, Ecole Polytechnique, CNRS, Inserm, Université Paris-Saclay, Palaiseau, France

(4) Inria, Université Paris-Saclay, Palaiseau, France

# These authors contributed equally to this work.

* florence.ruggiero@ens-lyon.fr, allain@lms.polytechnique.fr,

marie-claire.schanne-klein@polytechnique.edu

SUPPLEMENTARY TABLE

	Young mice				Old mice			
	Multiscale data	Mechanics only	SHG only	Total	Multiscale data	Mechanics only	SHG only	Total
WT	24	7	1	32	17	4	1	22
K14-COL5A1	9	7	0	16	8	3	0	11
Col5a2$^{pN/+}$	14	2	2	18	8	0	0	8
TOTAL				66				41

Supplementary table 1: Number of mice used for multiscale, mechanics only and SHG only measurements.

SUPPLEMENTARY FIGURES

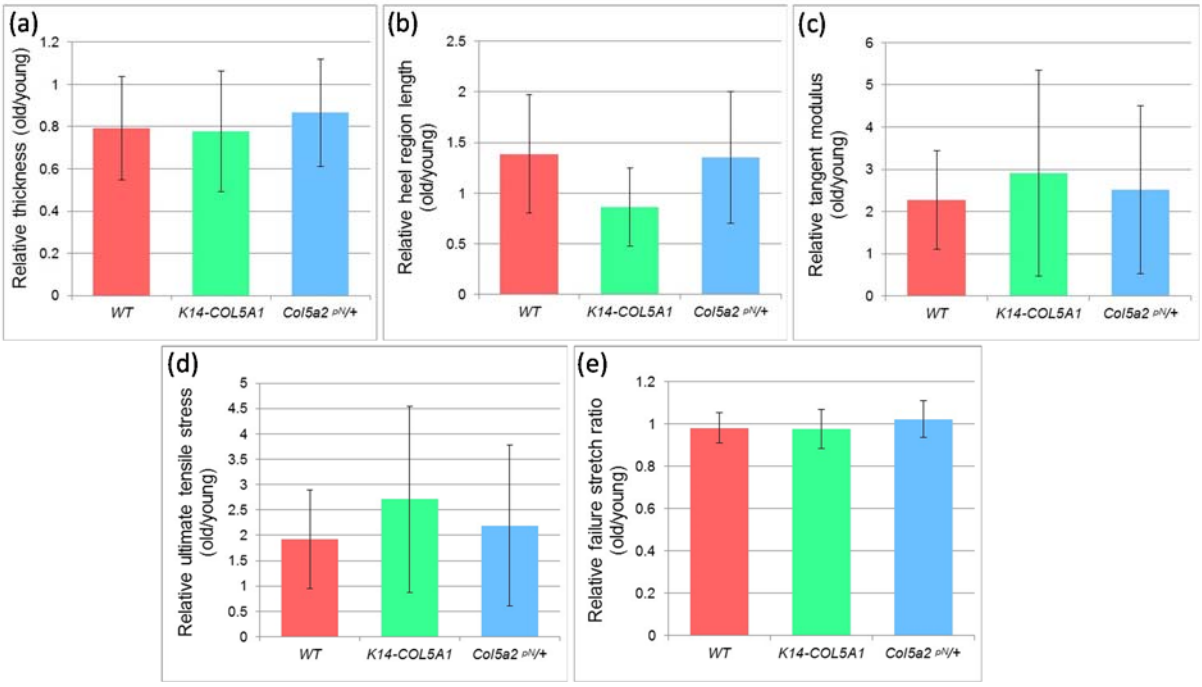

Figure S1: Mechanical data from old relatively to young mice, for WT (red), *K14-COL5A1* (green) and *Col5a2*$^{pN/+}$ mice (blue). These data are obtained as the ratio of the average value for old mice to the average value for young mice. The error bars correspond to standard deviation. (a) Relative skin thickness, (b) Relative length of the heel region, (c) Relative tangent modulus, (d) Relative ultimate tensile Stress, (e) Relative failure stretch ratio.

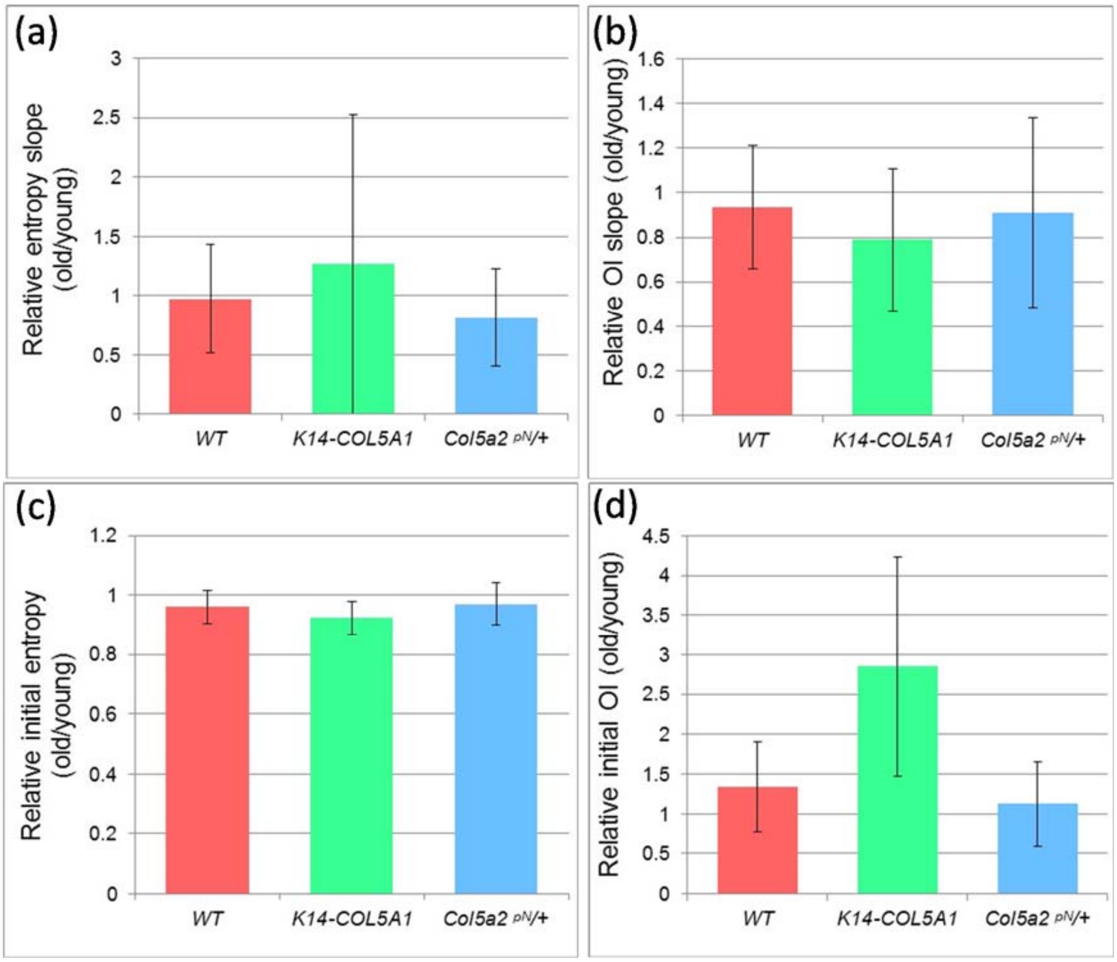

Figure S2: Microscopic data from old relatively to young mice, for WT (red), *K14-COL5A1* (green) and *Col5a2*$^{pN/+}$ mice (blue). These data are obtained as the ratio of the average value for old mice to the average value for young mice. The error bars correspond to standard deviation. Relative slope of the linear region of (a) the entropy variation and (b) the OI variation as a function of the stretch ratio. Relative initial value (no applied stretch) of (c) the entropy and (d) the OI.

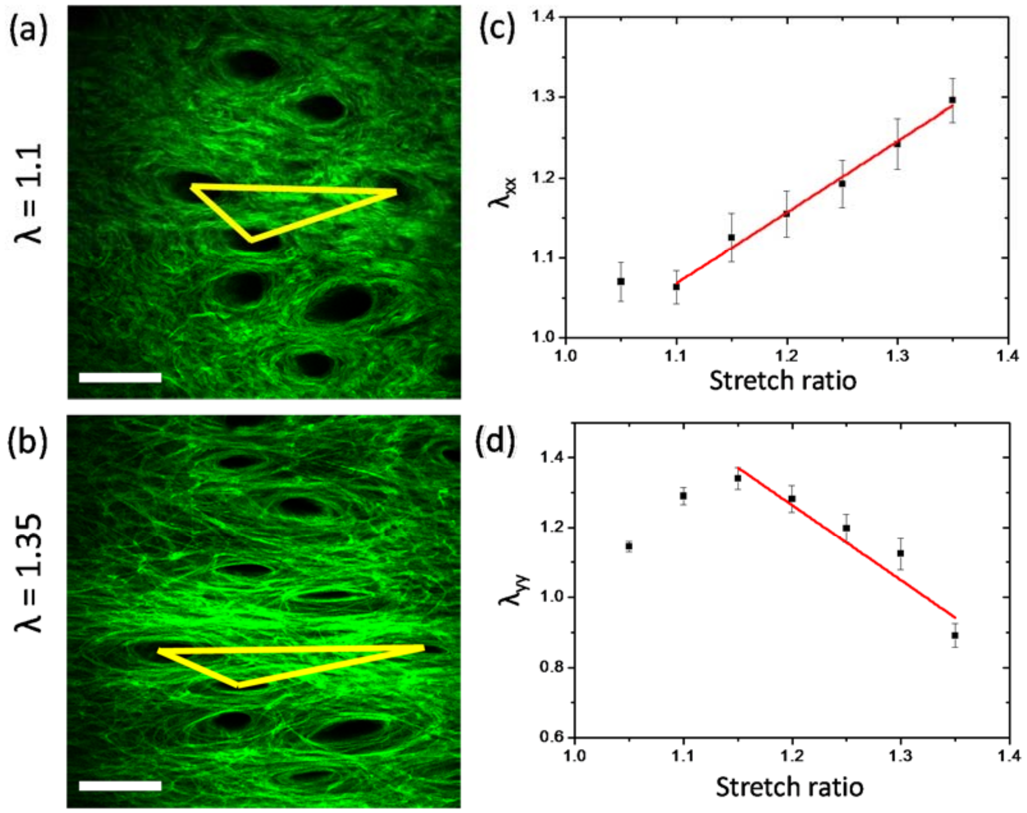

Figure S3: Local deformation of skin sample from an old WT mouse (same as in Figure 1 and 4). (a-b) SHG images of the skin of an old WT mouse at (a) 1.1 and at (b) 1.35 stretch ratio, as in Figure 1. The dark regions corresponding to the positions of hair follicles are used as endogenous tags to measure the local deformation tensor. The traction is along the horizontal direction. Scale bar: 100 µm. (c-d) Local stretch ratios along the (c) x and (d) y directions as a function of the applied stretch ratio. The red lines correspond to a linear fitting.

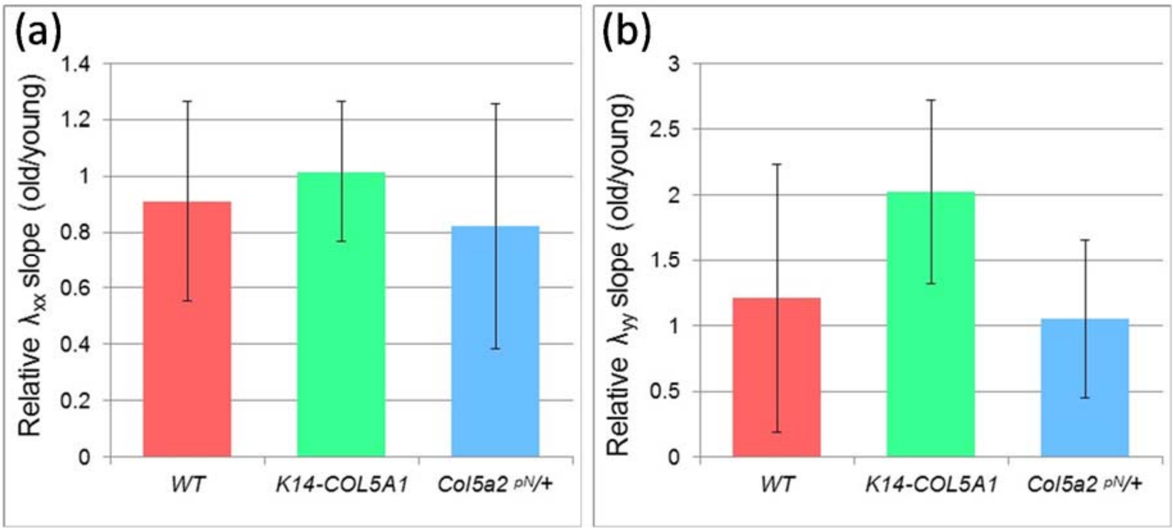

Figure S4: Local deformation data from old relatively to young mice for WT (red), *K14-COL5A1* (green) and *Col5a2*$^{pN/+}$ mice (blue). These data are obtained as the ratio of the averaged value for old mice to the average value for young mice. The error bars correspond to standard deviation. (a) Slope of λ_{xx}, (b) slope of λ_{yy}.

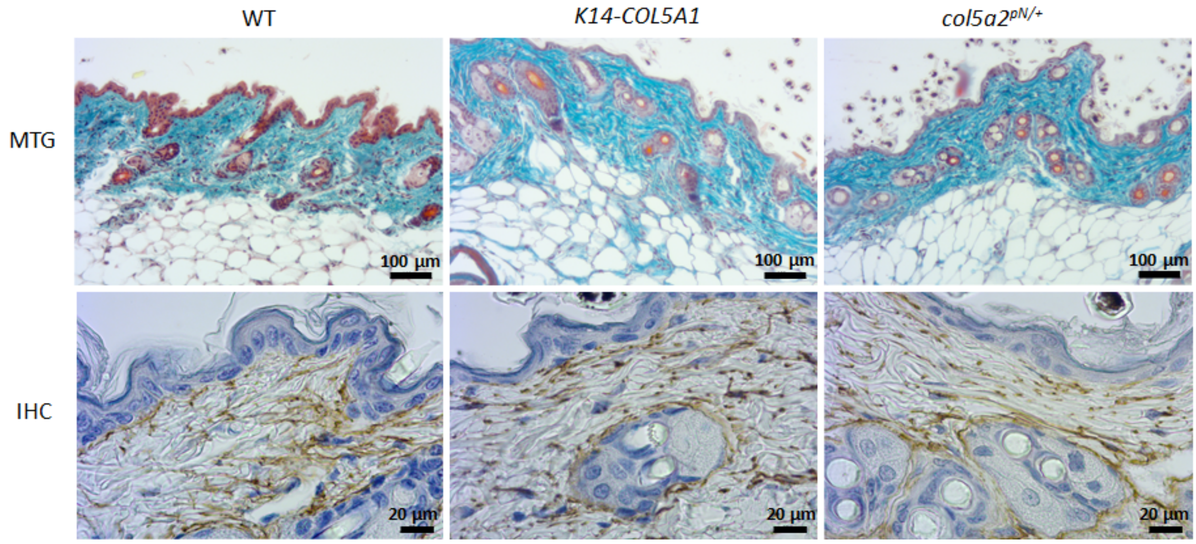

Figure S5: Masson's trichrome Goldner staining (MTG) and elastin immunohistochemistry (IHC) of 5 µm histological sections of WT, *K14-COL5A1* and *Col5a2*$^{pN/+}$ young mice. The overall structure of the skin and elastin deposits are qualitatively similar in all strains. Elastic fibers appear less abundant and thinner in the papillary dermis and upper reticular dermis compared to deep reticular dermis (accordingly, they are not seen in multiphoton images).
